# Test–retest reliability and familiarization effects of blood lactate accumulation metrics during 15‐s cycle ergometer sprints in athletes without specific cycling experience

**DOI:** 10.14814/phy2.71205

**Published:** 2026-11-06

**Authors:** Daniel Röhrs, Carl‐Maximilian Wagner, Nicolas Neumann, Rüdiger Reer, Tobias Schmidt, Michael Keiner

**Affiliations:** ^1^ Department of Sports and Exercise Medicine, Institute for Human Movement Science University of Hamburg Hamburg Germany; ^2^ Institute of Exercise, Sport and Health Leuphana University Lüneburg Lüneburg Germany; ^3^ MSH Medical School Hamburg Institute of Interdisciplinary Exercise Science and Sports Medicine Hamburg Germany; ^4^ Department of Sport Science German University of Health and Sport Ismaning Germany

**Keywords:** blood lactate, cycling, familiarization, lactate accumulation, sprint performance, test–retest reliability

## Abstract

This study evaluated the test–retest reliability of mechanical sprint outcomes, maximal blood lactate accumulation rate (*v*Lamax_blood_), and peak lactate accumulation rate (*v*Lapeak) during 15‐s cycle ergometer sprints in athletes without specific cycling experience, and assessed familiarization effects. Fifty active participants completed three maximal 15‐s sprints on separate days. Peak and mean power, blood lactate variables, the alactic time component (*t*_alac_), *v*Lamax_blood_, and *v*Lapeak were assessed. Relative reliability was quantified using intraclass correlation coefficients, and absolute reliability using standard error of measurement, typical error, coefficient of variation (CV), mean absolute percentage error, minimal detectable change (MDC), smallest worthwhile change (SWC), and Bland–Altman analysis. Mechanical variables showed high reliability (ICC = 0.93–0.99; CV = 1.75%–4.39%) but systematic increases across sessions, indicating familiarization. *v*Lamax_blood_ showed lower reliability (ICC = 0.75–0.92; CV = 10.69%–13.69%; MDC = 0.19–0.20 mmol·L^−1^·s^−1^), whereas vLapeak showed good relative reliability (ICC = 0.86–0.87) and lower measurement error (CV = 9.06%–10.10%; MDC = 0.11–0.12 mmol·L^−1^·s^−1^). For *v*Lamax_blood_, *v*Lapeak, and *t*_alac_, MDC exceeded SWC. At least one familiarization session appears advisable before baseline testing, and small within‐athlete changes in lactate‐accumulation metrics should be interpreted cautiously.

## 1 INTRODUCTION

Short high‐intensity efforts are relevant across many sports, including sprint disciplines, team sports, and endurance events with intermittent surges (Haugen et al., 2021; Thurlow et al., 2023). Their partial dependence on anaerobic glycolytic energy provision highlights the relevance of assessing anaerobic glycolytic capacity for performance diagnostics and training prescription (Gastin, 2001; Heck & Schulz, 2002; Yang et al., 2023). A commonly used surrogate measure of maximal glycolytic power is the maximal blood lactate accumulation rate (*v*Lamax_blood_), calculated as the post‐exercise increase in blood lactate concentration divided by the effective exercise time after subtraction of the alactic time component (*t*_alac_) (Mader, 1984; Wackerhage et al., 2025). Although *v*Lamax_blood_ may complement aerobic markers such as maximal oxygen uptake or lactate thresholds, its determination is influenced by several methodological factors, including sprint duration, exercise modality, baseline lactate concentration, post‐exercise blood sampling procedures, and the method used to estimate the alactic or phosphagenic time component (Langley et al., 2025; Quittmann, 2026; Röhrs et al., 2026). In particular, different approaches to determining the alactic time component can influence the calculated *v*Lamax_blood_ and its reliability (Meixner et al., 2024).

This methodological sensitivity is reflected in the current literature. A recent systematic review of 11 studies showed that test–retest reliability for *v*Lamax_blood_ varied substantially across exercise modalities and protocols, with intraclass correlation coefficients (ICC) ranging from 0.47 to 0.96 and coefficients of variation (CV) from 4.6% to 26.4% (Röhrs et al., 2026). This variation suggests that reliability is strongly influenced by protocol design, exercise modality, blood lactate sampling, and, in particular, the method used to estimate *t*_alac_, which is in line with a recent review emphasizing substantial methodological heterogeneity in *v*Lamax_blood_ testing (Quittmann, 2026). Moreover, ICCs and CVs primarily describe reliability at the group level and do not fully capture the magnitude of individual measurement error. Additional indices such as the mean absolute percentage error (MAPE), minimal detectable change (MDC), and Bland–Altman limits of agreement are therefore important for evaluating whether repeated *v*Lamax_blood_ measurements are sufficiently precise to detect meaningful within‐athlete changes (Atkinson & Nevill, 1998; Hopkins, 2000; Warneke et al., 2025). This is particularly relevant for longitudinal monitoring, because high relative reliability can coexist with substantial absolute measurement error; if this error exceeds physiologically meaningful changes, the test may fail to detect relevant adaptations despite appearing statistically reliable.

A further methodological consideration concerns the correction for *t*_alac_. Because *t*_alac_ is estimated from the sprint performance profile and enters the denominator of the *v*Lamax_blood_ calculation, variability in its determination may propagate into the resulting *v*Lamax_blood_ value. Recently, *v*Lapeak has been proposed as an alternative lactate‐accumulation metric that relates the exercise‐induced increase in blood lactate concentration to the complete sprint duration without correction for *t*_alac_ (Wackerhage et al., 2025). By omitting this estimated component, *v*Lapeak may reduce one potential source of calculation‐related variability. However, this simplification may come at the cost of physiological information captured by the *t*_alac_ correction. Whereas *v*Lamax_blood_ attempts to account for the initial phase of the sprint before peak power is reached, *v*Lapeak distributes the observed increase in blood lactate across the entire sprint duration. Consequently, *v*Lapeak should not necessarily be regarded as a direct substitute for *v*Lamax_blood_, but rather as a related, more directly calculated measure of lactate accumulation with potentially different physiological interpretation. The test–retest reliability and individual‐level measurement error of *v*Lapeak have not yet been established. Direct comparison of *v*Lamax_blood_ and *v*Lapeak within the same repeated‐sprint protocol may therefore clarify whether the increased computational simplicity of *v*Lapeak translates into improved measurement precision and how this should be weighed against the information omitted by removing the *t*_alac_ correction.

Measurement error may arise not only from biological day‐to‐day variation, but also from technical aspects such as lactate analyzers, ergometer setup, and the execution of maximal effort (Atkinson & Nevill, 1998). Maximal sprint testing is further influenced by neuromuscular coordination, pacing behavior, and motivation, making familiarization with the testing modality especially relevant (Driss & Vandewalle, 2013). This issue may be particularly relevant when athletes without specific cycling experience perform repeated cycle‐based sprint tests because repeated exposure to an unfamiliar task may induce systematic changes in performance due to familiarization or learning effects rather than physiological adaptation. Evidence from both cycling‐ and running‐based high‐intensity protocols indicates that such effects can influence performance outcomes, suggesting that derived physiological or metabolic variables may also be affected when they depend on the underlying mechanical output (Glaister et al., 2009; Ozkaya, 2013).

Recently, Sablain et al. (2025) examined the reliability of *v*Lamax_blood_ across four repeated 15‐s cycling sprints in 13 physically active men and reported good reliability for *v*Lamax_blood_ (ICC = 0.80; CV = 7%), whereas the reliability of *t*_alac_ was considerably lower (ICC = 0.25). While these findings provide important information for the repeatability of *v*Lamax_blood_, they were obtained in a small sample of 13 participants and did not comprehensively address individual‐level measurement error or familiarization effects. The present study extends this work by examining a larger sample and by providing a broader assessment of absolute measurement error and familiarization across repeated testing.

Against this background, an important research gap remains regarding the reliability and practical interpretability of lactate‐accumulation metrics in athletes without specific cycling experience. In particular, it is unclear to what extent familiarization affects repeated sprint performance and lactate outcomes, and whether *v*Lamax_blood_ is sufficiently stable and sensitive for use in longitudinal diagnostics in this population. Therefore, the primary aim of the present study was to investigate the test–retest reliability of *v*Lamax_blood_ derived from a 15‐s maximal cycle ergometer sprint across three repeated test sessions in participants without specific cycling experience, with particular focus on familiarization effects and the reliability of sprint power and lactate‐related outcomes. A secondary aim was to determine the test–retest reliability of *v*Lapeak and to examine whether omission of the *t*_alac_ correction was associated with lower absolute measurement error compared with *v*Lamax_blood_. Because testing was conducted on two cycle ergometer systems (SRM and Lode Excalibur Sport), potential device‐related influences were considered in the statistical analysis, providing additional information on the robustness of the reliability findings across different laboratory setups. It was hypothesized that (i) mechanical power would demonstrate excellent reliability but systematic improvements between sessions due to familiarization, whereas (ii) *v*Lamax_blood_ would show lower reliability, greater absolute error, and therefore limited sensitivity to detect small but meaningful changes, with only minor systematic learning effects, and (iii) *v*Lapeak would demonstrate lower absolute measurement error than *v*Lamax_blood_ due to the omission of the *t*_alac_ correction.

## 2 MATERIALS AND METHODS

This study used a test–retest design to examine the reliability of mechanical sprint outcomes and metabolic variables during repeated cycle sprint testing in recreationally active athletes without specific cycling experience. Each participant completed three identical testing sessions under standardized laboratory conditions, with 48 h between consecutive sessions. The primary variables were peak power output, mean power output, and *v*Lamax_blood_. *v*Lapeak was additionally evaluated as a secondary lactate‐accumulation metric.

### 2.1 Participants

Fifty active athletes without specific cycling experience were included in the study. Participants were recruited through local sports clubs, university networks, and personal contact with recreationally active individuals. Eligibility was assessed prior to inclusion based on self‐reported training background, health status, and cycling experience. Inclusion criteria were: (1) age ≥ 18 years, (2) self‐reported regular participation in recreational sport or exercise training for approximately 1 year or longer before study enrolment, (3) no structured cycling or cycle‐ergometer training within the previous 6 months, and (4) no contraindication to maximal exercise testing. Exclusion criteria were current musculoskeletal injury, known cardiovascular, metabolic, or neurological disease, acute illness at the time of testing, and any medical or orthopedic condition that could limit safe participation in maximal exercise testing. All participants were informed about the study procedures before testing and provided written informed consent. The study was approved by Ethics Committee of the Medical School Hamburg (MSH; approval number: MSH‐2023/287) and conducted in accordance with the Declaration of Helsinki. Participant characteristics are presented in Table 1.

**TABLE 1:** Participant characteristics.

Characteristic	Total (*n* = 50)	Lode (*n* = 17)	SRM (*n* = 33)
Age [years]	27 ± 7	27 ± 6	27 ± 7
Height [cm]	177.3 ± 9.4	176.6 ± 10.0	177.7 ± 9.2
Body mass [kg]	75.4 ± 11.6	73.8 ± 12.4	76.2 ± 11.2

### 2.2 Sample size estimation and justification

In line with recent recommendations for transparent sample size reporting in sport and exercise science (Abt et al., 2025), sample size estimation was reported for the primary reliability analysis of *v*Lamax_blood_ across three repeated test sessions. The required sample size was calculated using the ICC‐based approach described by Walter et al. (1998). Previous *v*Lamax_blood_ reliability studies reported heterogeneous reliability estimates, with ICCs ranging from 0.47 to 0.96 and a pooled ICC of approximately 0.84 (Adam et al., 2015; Harnish et al., 2023; Harnish et al., 2024; Held et al., 2023; Meixner et al., 2024; Nitzsche et al., 2018; Quittmann, Abel, et al., 2021; Quittmann et al., 2020; Quittmann, Schwarz, et al., 2021; Sablain et al., 2025; Wawer et al., 2020). Therefore, a conservative expected ICC of 0.85 was assumed for the sample size calculation. Assuming three repeated measurements per participant, a minimum acceptable ICC of 0.70, *α* = 0.05, and 95% statistical power, the required sample size was estimated to be approximately 50 participants. Accordingly, the target sample size was set at 50 participants, in line with Hopkins' recommendation for achieving reasonable precision in reliability estimates, and was achieved in the present study (Hopkins, 2000).

### 2.3 Procedures

All tests were performed at approximately the same time of day (±1.5 h) under standardized laboratory conditions in a temperature‐controlled environment (18°C–21°C). Participants were instructed to maintain their habitual diet and training routines, consume a carbohydrate‐rich meal approximately 2 h before each test, avoid strenuous exercise for 24 h before testing, and refrain from caffeine or other stimulants for at least 3 h prior to each session. Upon arrival, capillary blood samples were collected from the right earlobe to determine resting blood lactate concentration and analyzed using a laboratory‐based enzymatic‐amperometric lactate analyzer (Biosen C‐Line, EKF Diagnostics, Germany). Participants then completed a standardized 5‐min warm‐up at 1.5 W·kg^−1^ body mass, followed by 5 min of passive recovery. If pre‐test lactate concentration exceeded 2 mmol·L^−1^, the recovery period was extended until values fell below this threshold. Subsequently, participants performed a 15‐s all‐out sprint from a stationary start on an isokinetic cycle ergometer with cadence controlled at 130 rpm. Sprints were performed in a seated position, and saddle and handlebar positions were standardized and replicated across sessions. Strong verbal encouragement was provided throughout each effort to promote maximal performance. Two ergometer systems were used (SRM, Schoberer Rad Messtechnik GmbH, Germany; Lode Excalibur Sport, Lode BV, The Netherlands). Technical issues with the SRM ergometer required the use of the Lode Excalibur Sport for part of the sample. Each participant completed all three sessions on the same ergometer. The Lode Excalibur Sport has demonstrated high laboratory reliability, with coefficient of variation values ≤5% across test stages, whereas SRM systems are widely used as reference devices for cycling power measurement and served as the reference device in 31 validation studies included in a recent scoping review (Bouillod et al., 2022; Earnest et al., 2005). To account for potential device‐related influences, ergometer type was considered in the statistical analysis, and reliability outcomes were additionally evaluated separately for each device. Power output and cadence were recorded continuously at device‐specific sampling rates (10 Hz for SRM and 5 Hz for Lode). Following each sprint, participants remained seated while capillary blood samples were collected at 1‐min intervals until peak lactate concentration was identified. Peak lactate was defined as the highest measured post‐exercise value, and sampling was discontinued after two consecutive measurements showed a decline.

### 2.4 Calculations

Power output and cadence were recorded continuously and used to derive peak power output (P_peak_) and mean power output (P_mean_) over the 15‐s sprint. Both variables were reported as absolute values (W) and normalized to body mass (W·kg^−1^).

The maximal blood lactate accumulation rate (*v*Lamax_blood_) was calculated according to established procedures as (Heck et al., 2003):

$$v{\text{Lamax}}_{\text{blood}}=\frac{{\mathrm{La}}_{\text{peak}}-{\mathrm{La}}_{\text{rest}}}{t_{\text{exercise}}-t_{\text{alac}}}$$

where La_rest_ and La_peak_ represent resting and peak lactate concentrations, respectively, and *t*_exercise_ is the sprint duration (15 s). Consistent with sprint‐based approaches, *t*_alac_ was operationally defined as the time to peak power output derived from the power–time curve. No mathematical curve‐fitting model was applied. Peak power was defined as the highest recorded power value during the sprint, and *t*_alac_ as the elapsed time from sprint onset to the corresponding time point. No additional device‐specific time‐delay correction was applied to the data.

In addition, *v*Lapeak was calculated as:

$$v\text{Lapeak}=\frac{{\left[\mathrm{La}\right]}_{\text{peak}}-{\left[\mathrm{La}\right]}_{\text{rest}}}{t_{\text{exercise}}}$$

### 2.5 Statistics

Data are presented as mean ± SD. Normality of residuals was assessed using the Shapiro–Wilk test. Systematic differences across the three repeated measurements were evaluated using repeated‐measures ANOVA with Greenhouse–Geisser correction where appropriate. Effect sizes for ANOVA effects were reported as partial eta squared (ηp^2^) and interpreted as small (≥0.01), medium (≥0.06), or large (≥0.14). When a significant main effect was observed, Bonferroni‐adjusted pairwise comparisons were performed to identify differences between individual time points. Effect sizes for pairwise comparisons were calculated as Cohen's *d*z for dependent samples and were interpreted as small (0.20), moderate (0.50), and large (0.80) (Cohen, 1988).

As multivariate omnibus analyses, complete‐case Hotelling's *T* ^2^ tests based on change scores were performed separately for each ergometer and variable block. The mechanical block included peak power and mean power, whereas the lactate/*v*Lamax_blood_ block included peak blood lactate, *t*_alac_, *v*Lamax_blood_, and delta lactate (Δ lactate). These analyses were used as preliminary multivariate tests of systematic changes across repeated measurements, followed by univariate repeated‐measures ANOVAs for individual outcomes. Potential differences in changes over time between ergometer groups were examined using mixed ANOVAs with time as a within‐subject factor and ergometer as a between‐subject factor. Ergometer main effects were interpreted descriptively because ergometer allocation was a between‐subject factor. In the absence of significant time × ergometer interactions, Bonferroni‐adjusted pairwise comparisons of the overall time effect were used to characterize systematic changes across sessions.

Associations between *v*Lamax_blood_ and *v*Lapeak were examined separately for each test session using Spearman rank correlations. Reliability across repeated measurements was assessed using both relative and absolute indices. Relative reliability was quantified using intraclass correlation coefficients derived from a two‐way mixed‐effects model with absolute agreement for single measurements (ICC(A,1)). ICC values are reported with 95% confidence intervals and were interpreted as poor (<0.50), moderate (0.50–0.75), good (0.75–0.90), or excellent (>0.90) (Koo & Li, 2016). Absolute reliability was evaluated using the standard error of measurement (SEM), typical error (TE), coefficient of variation (CV), mean absolute error (MAE), and mean absolute percentage error (MAPE). SEM was calculated as SD × √(1 − ICC), TE as the SD of the difference scores divided by √2, and CV as TE expressed relative to the mean. MAE was calculated as the mean absolute difference between repeated measurements, and MAPE as the mean absolute percentage difference relative to the corresponding pairwise mean value. Minimal detectable change (MDC) was calculated as 1.96 × √2 × SEM, and smallest worthwhile change (SWC) as 0.2 × the between‐subject SD. Bootstrapped 95% confidence intervals based on 2000 resamples were calculated for the absolute reliability indices. To support practical interpretation, MDC and SWC were compared, with MDC values exceeding the SWC indicating limited sensitivity to detect small but practically meaningful changes (Atkinson & Nevill, 1998; Hopkins, 2000). Agreement between repeated measurements was further examined using Bland–Altman analysis by calculating mean bias and 95% limits of agreement (Bland & Altman, 1986; Bland & Altman, 1999). For all Bland–Altman analyses, differences were calculated as the later test session minus the earlier test session (i.e., T2–T1, T3–T1, and T3–T2).

Statistical significance was set at *p* < 0.05. All statistical analyses were performed using R (Version 4.5.0; R Foundation for Statistical Computing, Vienna, Austria).

## 3 RESULTS

Descriptive values for all mechanical and metabolic variables across the three test sessions, presented separately for each ergometer, are shown in Table 2. Shapiro–Wilk tests of model residuals indicated deviations from normality for several variables, including peak power, mean power, relative power outputs, *v*Lamax_blood_, and lactate variables, whereas *t*_alac_ did not significantly deviate from normality. The planned ANOVA‐based analyses were retained. Mauchly's test indicated violations of sphericity for peak power and relative peak power on both ergometers, for mean power on the SRM ergometer, and for resting lactate on the SRM ergometer; therefore, Greenhouse–Geisser‐corrected degrees of freedom are reported for these outcomes.

**TABLE 2:** Descriptive values for mechanical and metabolic variables across the three test sessions, presented separately for each ergometer.

Variable	Ergometer	T1	T2	T3	Time effect, *F* (df1, df2)	ηp^2^
*Mechanical variables*						
P_peak_ [W]	Lode (*n* = 17)	755 ± 217	768 ± 214	774 ± 223	4.32 (1.36, 21.8)*	0.213
	SRM (*n* = 33)	868 ± 239	896 ± 229	898 ± 232	9.26 (1.51, 48.3)*	0.224
Relative peak power [W·kg^−1^]	Lode (*n* = 17)	10.08 ± 1.41	10.27 ± 1.34	10.33 ± 1.45	4.81 (1.37, 21.9)*	0.231
	SRM (*n* = 33)	11.23 ± 1.94	11.62 ± 1.74	11.63 ± 1.84	9.54 (1.54, 49.1)*	0.230
P_mean_ [W]	Lode (*n* = 17)	646 ± 191	657 ± 186	661 ± 189	5.04 (2, 32)*	0.239
	SRM (*n* = 33)	702 ± 185	717 ± 187	721 ± 194	3.62 (1.61, 51.4)*	0.102
Relative mean power [W·kg^−1^]	Lode (*n* = 17)	8.65 ± 1.27	8.77 ± 1.17	8.82 ± 1.21	5.84 (2, 32)*	0.267
	SRM (*n* = 33)	9.09 ± 1.53	9.30 ± 1.50	9.33 ± 1.62	3.37 (2, 64)*	0.095
*Metabolic variables*						
Resting lactate [mmol·L^−1^]	Lode (*n* = 17)	0.93 ± 0.16	0.92 ± 0.14	0.93 ± 0.17	0.04 (2, 32)	0.003
	SRM (*n* = 33)	0.98 ± 0.32	1.14 ± 0.71	1.10 ± 0.39	1.46 (1.33, 42.6)	0.044
La_rest_ [mmol·L^−1^]	Lode (*n* = 17)	1.15 ± 0.19	1.13 ± 0.13	1.14 ± 0.13	0.48 (2, 32)	0.029
	SRM (*n* = 33)	1.23 ± 0.43	1.19 ± 0.38	1.26 ± 0.40	0.57 (2, 64)	0.017
La_peak_ [mmol·L^−1^]	Lode (*n* = 17)	8.13 ± 1.94	8.45 ± 1.89	8.23 ± 1.86	1.06 (2, 32)	0.062
	SRM (*n* = 33)	6.69 ± 1.71	6.93 ± 1.46	6.98 ± 1.64	2.15 (2, 64)	0.063
*t*_alac_ [s]	Lode (*n* = 17)	4.9 ± 1.3	4.7 ± 1.1	4.9 ± 0.6	0.67 (2, 32)	0.040
	SRM (*n* = 33)	4.0 ± 1.1	3.9 ± 0.9	3.8 ± 1.0	0.75 (2, 64)	0.023
*v*Lamax_blood_ [mmol·L^−1^·s^−1^]	Lode (*n* = 17)	0.71 ± 0.27	0.71 ± 0.26	0.69 ± 0.25	0.59 (2, 32)	0.035
	SRM (*n* = 33)	0.50 ± 0.15	0.52 ± 0.12	0.51 ± 0.14	0.66 (2, 64)	0.020
*v*Lapeak [mmol·L^−1^·s^−1^]	Lode (*n* = 17)	0.46 ± 0.12	0.49 ± 0.12	0.47 ± 0.12	1.23 (2, 32)	0.071
	SRM (*n* = 33)	0.36 ± 0.11	0.38 ± 0.10	0.38 ± 0.10	2.35 (2, 64)	0.068
Δ lactate [mmol·L^−1^]	Lode (*n* = 17)	6.97 ± 1.86	7.32 ± 1.83	7.09 ± 1.80	1.23 (2, 32)	0.071
	SRM (*n* = 33)	5.47 ± 1.66	5.74 ± 1.45	5.73 ± 1.57	2.35 (2, 64)	0.068

*Note*: Values are presented as mean ± SD. Time effects were tested using repeated‐measures ANOVAs separately for each ergometer. Greenhouse–Geisser‐corrected degrees of freedom are reported where sphericity was violated.

Abbreviations: ANOVA, analysis of variance; La_peak_, peak post‐exercise blood lactate concentration; La_rest_, pre‐sprint blood lactate concentration used for the calculation; P_mean_, mean power output; P_peak_, peak power output; resting lactate, lactate concentration measured upon arrival before warm‐up; SD, standard deviation; T1–T3, test sessions 1–3; *t*_alac_, alactic time component; *v*Lamax_blood_, maximal blood lactate accumulation rate; *v*Lapeak, peak lactate accumulation rate calculated over the complete sprint duration without correction for *t*_alac_; Δ lactate, increase in blood lactate concentration from La_rest_ to La_peak_; ηp^2^, partial eta squared.

* *p* < 0.05.

Complete‐case multivariate omnibus tests showed a significant time effect for the mechanical block in both ergometer groups (Lode: *F*(4,13) = 3.80, *p* = 0.029; SRM: *F*(4,29) = 3.32, *p* = 0.024). In contrast, no significant multivariate time effect was observed for the lactate/*v*Lamax_blood_ block in either ergometer group (Lode: *F*(8,9) = 2.03, *p* = 0.156; SRM: *F*(8,25) = 1.91, *p* = 0.104). The corresponding univariate repeated‐measures ANOVA results, including *F* values, degrees of freedom, and partial eta squared, are presented in Table 2. No significant time × ergometer interactions were observed for the mechanical outcomes (all *p* ≥ 0.406). Overall Bonferroni‐adjusted pairwise comparisons showed increases in peak and relative peak power from T1 to T2 and from T1 to T3, whereas mean and relative mean power increased from T1 to T3. No significant differences were observed from T2 to T3. Effect sizes for the significant pairwise comparisons ranged from *d*z = 0.475 to 0.611.

Relative and absolute reliability indices for mechanical sprint variables and metabolic variables, separated by ergometer, are presented in Tables 3 and 4. Across ergometers, mechanical variables showed higher ICC values and lower relative error indices than metabolic variables. *v*Lapeak demonstrated good relative reliability on both ergometers and lower relative error than *v*Lamax_blood_, whereas *t*_alac_ showed the least favorable reliability characteristics. No significant time effect was observed for *v*Lapeak on either ergometer. For *v*Lamax_blood_, *v*Lapeak, and *t*_alac_, MDC values were higher than the corresponding SWC values.

**TABLE 3:** Relative and absolute reliability indices for mechanical sprint variables, presented separately for each ergometer.

Variable	Ergometer	*n*	ICC	SEM	TE	MAE	CV [%]	MAPE [%]	MDC	SWC
P_peak_ [W]	Lode (*n* = 17)	17	0.990 (0.977–0.996)	20.84 (15.01–24.37)	18.68 (10.36–24.58)	21.57 (15.10–29.53)	2.44 (1.40–3.20)	2.82 (2.07–3.64)	57.77 (42.18–67.63)	43.49 (31.83–51.29)
	SRM (*n* = 33)	33	0.977 (0.952–0.988)	35.43 (27.93–42.23)	31.27 (22.39–37.80)	34.59 (24.69–45.17)	3.52 (2.53–4.38)	4.06 (2.82–5.45)	98.21 (77.24–116.16)	46.40 (36.36–55.14)
Relative peak power [W·kg^−1^]	Lode (*n* = 17)	17	0.963 (0.912–0.986)	0.26 (0.19–0.30)	0.23 (0.13–0.31)	0.28 (0.21–0.38)	2.30 (1.34–2.98)	2.82 (2.07–3.70)	0.73 (0.53–0.85)	0.28 (0.20–0.32)
	SRM (*n* = 33)	33	0.935 (0.872–0.968)	0.47 (0.39–0.52)	0.41 (0.28–0.52)	0.45 (0.33–0.59)	3.58 (2.44–4.53)	4.06 (2.87–5.40)	1.29 (1.08–1.46)	0.36 (0.30–0.41)
P_mean_ [W]	Lode (*n* = 17)	17	0.995 (0.988–0.998)	12.80 (9.39–14.85)	11.73 (8.49–13.85)	14.47 (10.98–18.04)	1.79 (1.32–2.11)	2.32 (1.72–2.88)	35.48 (25.49–40.98)	37.63 (27.90–43.58)
	SRM (*n* = 33)	33	0.970 (0.947–0.984)	32.27 (25.63–37.51)	30.91 (23.65–35.89)	32.69 (24.36–41.58)	4.33 (3.33–5.16)	4.89 (3.63–6.56)	89.46 (70.00–105.95)	37.46 (29.75–43.84)
Relative mean power [W·kg^−1^]	Lode (*n* = 17)	17	0.980 (0.949–0.992)	0.17 (0.12–0.20)	0.15 (0.11–0.18)	0.20 (0.15–0.24)	1.75 (1.29–2.06)	2.32 (1.74–2.89)	0.47 (0.35–0.54)	0.24 (0.17–0.28)
	SRM (*n* = 33)	33	0.926 (0.871–0.960)	0.42 (0.35–0.48)	0.41 (0.31–0.48)	0.43 (0.32–0.54)	4.39 (3.30–5.27)	4.89 (3.55–6.48)	1.16 (0.96–1.33)	0.30 (0.24–0.35)

*Note*: Values in parentheses indicate 95% confidence intervals. SEM, TE, MAE, MDC, and SWC are expressed in the units of the respective variable. CV and MAPE are expressed as percentages.

Abbreviations: CV, coefficient of variation; ICC, intraclass correlation coefficient based on a two‐way mixed‐effects model with absolute agreement for single measurements (ICC(A,1)); MAE, mean absolute error; MAPE, mean absolute percentage error; MDC, minimal detectable change; *n*, number of participants; P_mean_, mean power output; P_peak_, peak power output; SEM, standard error of measurement; SWC, smallest worthwhile change; TE, typical error.

**TABLE 4:** Relative and absolute reliability indices for metabolic variables, presented separately for each ergometer.

Variable	Ergometer	*n*	ICC	SEM	TE	MAE	CV [%]	MAPE [%]	MDC	SWC
Resting lactate [mmol·L^−1^]	Lode (*n* = 17)	17	0.833 (0.674–0.930)	0.06 (0.04–0.08)	0.06 (0.04–0.08)	0.07 (0.05–0.09)	6.94 (4.50–8.05)	7.33 (5.64–9.17)	0.17 (0.10–0.22)	0.03 (0.02–0.04)
	SRM (*n* = 33)	33	0.325 (0.113–0.546)	0.41 (0.20–0.64)	0.40 (0.19–0.61)	0.32 (0.21–0.50)	37.23 (18.38–51.38)	26.52 (20.66–33.47)	1.15 (0.54–1.76)	0.07 (0.03–0.11)
La_rest_ [mmol·L^−1^]	Lode (*n* = 17)	17	0.753 (0.543–0.892)	0.07 (0.06–0.09)	0.08 (0.06–0.09)	0.09 (0.07–0.11)	6.69 (4.90–7.83)	7.84 (6.21–9.54)	0.21 (0.16–0.24)	0.03 (0.02–0.03)
	SRM (*n* = 33)	33	0.644 (0.467–0.788)	0.24 (0.21–0.26)	0.24 (0.19–0.28)	0.26 (0.21–0.32)	19.63 (15.29–23.39)	21.89 (17.67–26.37)	0.66 (0.57–0.73)	0.07 (0.06–0.08)
La_peak_ [mmol·L^−1^]	Lode (*n* = 17)	17	0.883 (0.762–0.952)	0.64 (0.41–0.79)	0.64 (0.46–0.77)	0.74 (0.54–0.94)	7.77 (5.47–9.46)	9.20 (6.75–11.79)	1.77 (1.13–2.20)	0.36 (0.22–0.46)
	SRM (*n* = 33)	33	0.854 (0.759–0.919)	0.61 (0.49–0.69)	0.60 (0.47–0.72)	0.67 (0.54–0.82)	8.78 (6.63–10.50)	10.57 (8.18–13.13)	1.69 (1.36–1.93)	0.31 (0.24–0.36)
*t*_alac_ [s]	Lode (*n* = 17)	17	0.493 (0.202–0.749)	0.72 (0.47–0.90)	0.73 (0.43–0.92)	0.59 (0.27–0.92)	15.12 (9.14–19.29)	12.31 (5.49–19.40)	2.00 (1.31–2.48)	0.17 (0.10–0.21)
	SRM (*n* = 33)	33	0.408 (0.195–0.616)	0.77 (0.64–0.88)	0.77 (0.62–0.89)	0.87 (0.72–1.03)	19.86 (15.86–23.44)	23.21 (18.99–28.26)	2.13 (1.80–2.43)	0.16 (0.12–0.19)
*v*Lamax_blood_ [mmol·L^−1^·s^−1^]	Lode (*n* = 17)	17	0.919 (0.831–0.967)	0.07 (0.04–0.10)	0.08 (0.04–0.10)	0.08 (0.05–0.11)	10.69 (6.17–14.16)	15.35 (7.99–25.24)	0.20 (0.11–0.28)	0.05 (0.02–0.07)
	SRM (*n* = 33)	33	0.745 (0.600–0.853)	0.07 (0.06–0.08)	0.07 (0.05–0.08)	0.07 (0.06–0.09)	13.69 (10.42–16.10)	14.78 (11.38–18.35)	0.19 (0.16–0.22)	0.02 (0.02–0.03)
*v*Lapeak [mmol·L^−1^·s^−1^]	Lode (*n* = 17)	17	0.871 (0.741–0.946)	0.04 (0.03–0.05)	0.04 (0.03–0.05)	0.05 (0.04–0.06)	9.06 (6.21–11.18)	10.89 (7.95–13.93)	0.12 (0.07–0.15)	0.02 (0.01–0.03)
	SRM (*n* = 33)	33	0.861 (0.770–0.923)	0.04 (0.03–0.04)	0.04 (0.03–0.04)	0.04 (0.03–0.05)	10.10 (7.75–12.14)	12.16 (9.21–15.62)	0.11 (0.09–0.12)	0.02 (0.02–0.02)
Δ lactate [mmol·L^−1^]	Lode (*n* = 17)	17	0.871 (0.741–0.946)	0.64 (0.41–0.81)	0.65 (0.45–0.78)	0.74 (0.54–0.96)	9.06 (6.15–11.28)	10.89 (8.16–13.98)	1.79 (1.15–2.22)	0.35 (0.21–0.44)
	SRM (*n* = 33)	33	0.861 (0.770–0.923)	0.58 (0.47–0.66)	0.57 (0.45–0.67)	0.62 (0.49–0.76)	10.10 (7.80–12.10)	12.16 (9.03–15.48)	1.60 (1.32–1.84)	0.30 (0.24–0.34)

*Note*: Values in parentheses indicate 95% confidence intervals. SEM, TE, MAE, MDC, and SWC are expressed in the units of the respective variable. CV and MAPE are expressed as percentages.

Abbreviations: CV, coefficient of variation; ICC, intraclass correlation coefficient based on a two‐way mixed‐effects model with absolute agreement for single measurements (ICC(A,1)); La_peak_, peak post‐exercise blood lactate concentration; La_rest_, pre‐sprint blood lactate concentration used for the calculation; MAE, mean absolute error; MAPE, mean absolute percentage error; MDC, minimal detectable change; *n*, number of participants; Resting lactate, blood lactate concentration measured upon arrival before warm‐up; SEM, standard error of measurement; SWC, smallest worthwhile change; *t*_alac_, alactic time component; TE, typical error; *v*Lamax_blood_, maximal blood lactate accumulation rate; *v*Lapeak, peak lactate accumulation rate calculated over the complete sprint duration without correction for *t*_alac_; Δ lactate, increase in blood lactate concentration from La_rest_ to La_peak_.

*v*Lamax_blood_ and *v*Lapeak were strongly correlated at each test session (T1: *r* = 0.829; T2: *r* = 0.836; T3: *r* = 0.798; all *p* < 0.001). Agreement analyses for *v*Lamax_blood_, *v*Lapeak, and *t*_alac_ are illustrated in Figures 1, 2, 3. Bland–Altman analysis showed small mean bias values across repeated measurements for *v*Lamax_blood_, *v*Lapeak and *t*_alac_. For *v*Lamax_blood_, mean bias ranged from −0.013 to 0.011 mmol·L^−1^·s^−1^, with limits of agreement from approximately −0.207 to 0.220 mmol·L^−1^·s^−1^ (Figure 1). For *v*Lapeak, mean bias ranged from −0.006 to 0.020 mmol·L^−1^·s^−1^ across the three pairwise comparisons (Figure 2). For *t*_alac_, mean bias ranged from −0.145 to −0.011 s, while the limits of agreement were markedly wider, ranging from approximately −2.328 to 2.100 s (Figure 3). Bland–Altman plots for peak power output and mean power output showed the corresponding bias and 95% limits of agreement across T2–T1, T3–T1, and T3–T2 comparisons (Figures 4 and 5).

**FIGURE 1:**
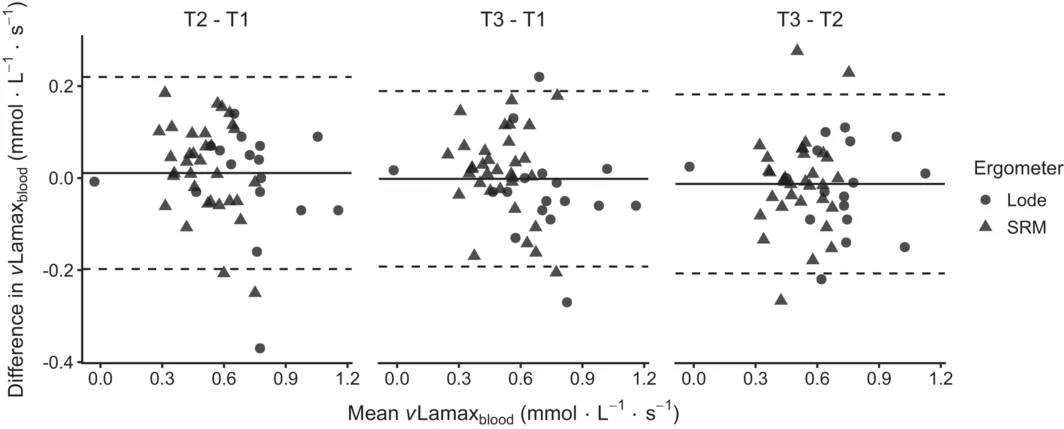
Bland–Altman plots showing agreement for *v*Lamax_blood_ between repeated test sessions: T2–T1, T3–T1, and T3–T2. Solid lines indicate mean bias and dashed lines indicate the 95% limits of agreement. Symbols distinguish between ergometer systems. Each panel includes *n* = 50 participants (Lode, *n* = 17; SRM, *n* = 33).

**FIGURE 2:**
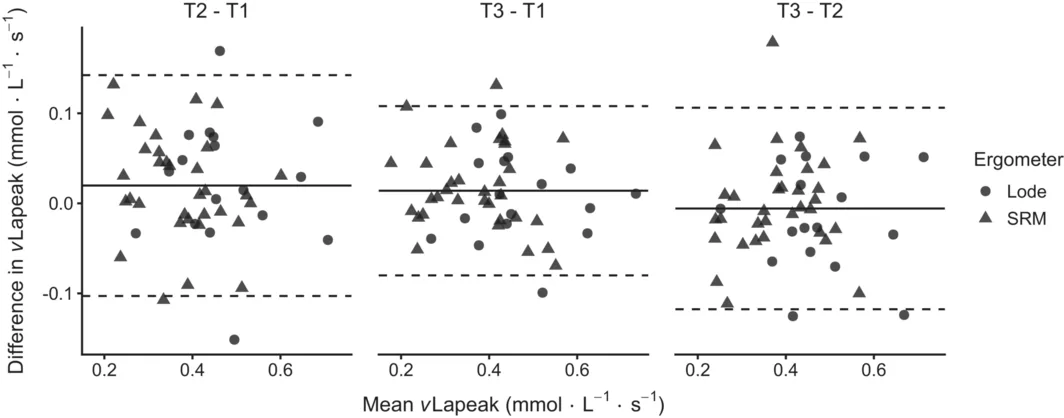
Bland–Altman plots showing agreement for *v*Lapeak between repeated test sessions: T2–T1, T3–T1, and T3–T2. Solid lines indicate mean bias and dashed lines indicate the 95% limits of agreement. Symbols distinguish between ergometer systems. Each panel includes *n* = 50 participants (Lode, *n* = 17; SRM, *n* = 33).

**FIGURE 3:**
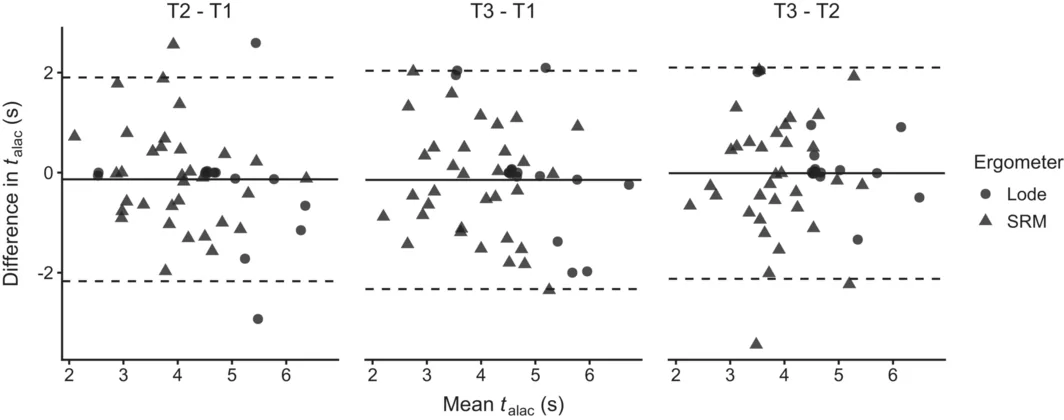
Bland–Altman plots showing agreement for *t*_alac_ between repeated test sessions: T2–T1, T3–T1, and T3–T2. Solid lines indicate mean bias and dashed lines indicate the 95% limits of agreement. Symbols distinguish between ergometer systems. Each panel includes *n* = 50 participants (Lode, *n* = 17; SRM, *n* = 33).

**FIGURE 4:**
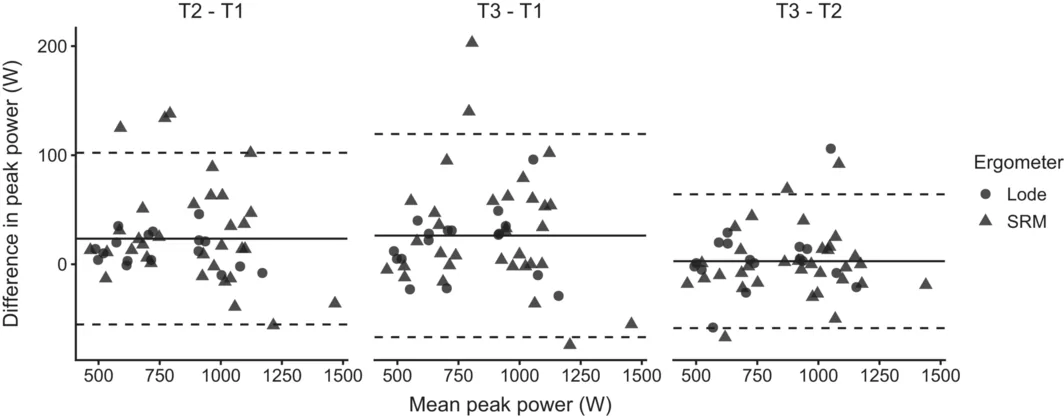
Bland–Altman plots showing agreement for peak power output between repeated test sessions: T2–T1, T3–T1, and T3–T2. Solid lines indicate mean bias and dashed lines indicate the 95% limits of agreement. Symbols distinguish between ergometer systems. Each panel includes *n* = 50 participants (Lode, *n* = 17; SRM, *n* = 33).

**FIGURE 5:**
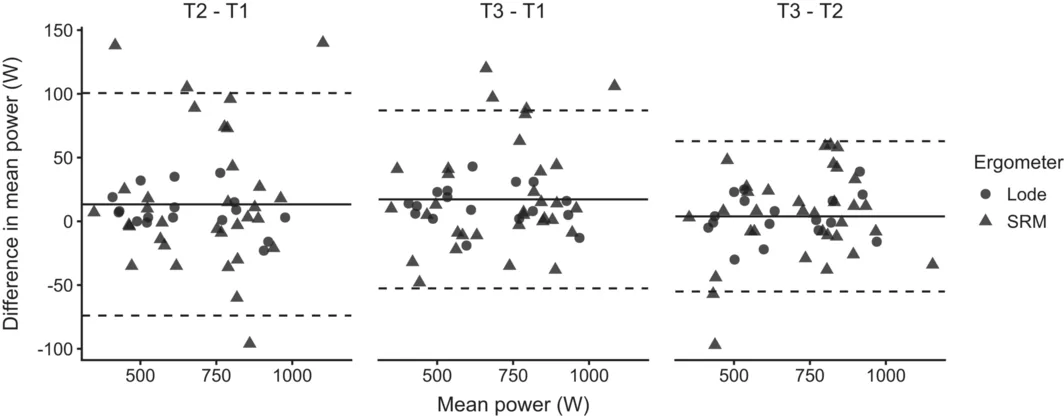
Bland–Altman plots showing agreement for mean power output between repeated test sessions: T2–T1, T3–T1, and T3–T2. Solid lines indicate mean bias and dashed lines indicate the 95% limits of agreement. Symbols distinguish between ergometer systems. Each panel includes *n* = 50 participants (Lode, *n* = 17; SRM, *n* = 33).

## 4 DISCUSSION

This study demonstrated a clear divergence between directly measured mechanical sprint outcomes and metabolic variables during repeated 15‐s cycle sprint testing in athletes without specific cycling experience. Peak and mean power showed high relative reliability and comparatively low absolute error, whereas *v*Lamax_blood_ and particularly *t*_alac_ showed larger within‐subject variability. *v*Lapeak showed good relative reliability on both ergometers and lower measurement error than *v*Lamax_blood_, although its relative reliability was not consistently higher. Mechanical power also increased across repeated sessions, while *v*Lamax_blood_, *v*Lapeak, and *t*_alac_ remained statistically unchanged over time. Accordingly, directly measured sprint power and calculated lactate outcomes require different interpretative thresholds in longitudinal monitoring. Potential device‐related influences were also considered, as testing was performed on two ergometer systems.

Mechanical sprint variables showed consistently high ICC values together with low TE, CV, MAE, and MAPE values. The low absolute error supports the reproducibility of mechanical sprint performance across repeated measurements, with only limited within‐athlete variation. Such a pattern supports the use of these variables for repeated performance monitoring under standardized test conditions (Atkinson & Nevill, 1998; Hopkins, 2000). The present findings are in line with previous work showing that peak and mean power during short maximal cycling sprints can be assessed with high reproducibility when test procedures are standardized and maximal effort is achieved (Driss & Vandewalle, 2013; Meixner et al., 2024). A likely explanation is that mechanical sprint outcomes are derived directly from ergometer data recorded during the sprint (Driss & Vandewalle, 2013). In contrast, *v*Lamax_blood_ requires additional post‐exercise blood sampling and calculation steps, which may increase its susceptibility to biological, procedural, analytical, and calculation‐related variability. In addition, blood lactate concentration reflects the balance between lactate appearance and removal and may therefore be influenced by factors such as muscle recruitment, training status, oxidative capacity, and lactate transport and clearance (Benitez‐Munoz & Cupeiro, 2025; Goodwin et al., 2007; Quittmann, 2026).

The systematic increase in mechanical power points to a familiarization effect despite high relative reliability. Peak power and relative peak power increased significantly from T1 to T2 and from T1 to T3, whereas no further significant increase was observed from T2 to T3. Mean power and relative mean power showed a smaller familiarization effect, with significant differences only between T1 and T3. The corresponding effect sizes for the significant pairwise comparisons ranged from *d*z = 0.475 to 0.611, indicating small to moderate improvements. Given that the participants had no specific cycling experience, these changes are unlikely to reflect physiological adaptation and are more plausibly explained by familiarization with the task. A particularly relevant comparison is provided by Meixner et al. (2024), who assessed reliability across three repeated 15‐s all‐out cycling sprints in 50 experienced cyclists and treated the first test as a familiarization trial. Their participants had more than 3 years of regular cycling experience (>2 sessions per week), whereas the present study specifically included athletes without recent structured cycling experience. This difference in cycling‐specific experience may increase the importance of familiarization in the present population and may partly explain the systematic improvements in mechanical power observed after the first exposure. Previous studies have shown that repeated exposure to cycle‐based performance testing can improve the reproducibility of power output and, in some settings, increase peak power during early trials, supporting the use of familiarization before baseline assessment (Abbiss et al., 2008; Kohler et al., 2010).

The size of these familiarization‐related changes is important, as it partly overlaps with improvements reported after short‐term training interventions targeting mechanical sprint performance. Previous cycling‐based interventions have reported increases in peak or mean sprint power of approximately 4%–12%, depending on the training stimulus, population, and test protocol (Hazell et al., 2010; Kristoffersen et al., 2019; Ronnestad et al., 2010). Therefore, using the first test as the sole baseline measure may lead to an overestimation of training‐induced changes, particularly when expected intervention effects are small.

After familiarization, however, the low random error of the mechanical variables suggests that the protocol is likely to be sufficiently sensitive to detect meaningful changes in sprint power. The absence of significant differences between T2 and T3 for peak power and relative peak power indicates that the largest familiarization effect occurred after the first exposure, although additional stabilization with further practice cannot be ruled out. For athletes without specific cycling experience, the first sprint test should therefore not be used as the sole baseline assessment. At least one familiarization session appears advisable before baseline values are used for monitoring, while a third baseline trial may be useful when high precision is required.

Compared with the mechanical sprint outcomes, *v*Lamax_blood_ and *t*_alac_ showed less favorable reliability characteristics. Although *v*Lamax_blood_ was more reliable than *t*_alac_, absolute error remained clearly larger than for mechanical power variables. This has important implications for longitudinal monitoring. In the present study, the MDC for *v*Lamax_blood_ was approximately 0.19–0.20 mmol·L^−1^·s^−1^, whereas the SWC was clearly lower. The corresponding SEM was approximately 0.07 mmol·L^−1^·s^−1^, while TE and MAE were approximately 0.07–0.08 mmol·L^−1^·s^−1^ across ergometer groups, indicating changes of this magnitude may already fall within the expected measurement variability of the test. Direct comparisons with previous studies are limited because MDC has rarely been reported for *v*Lamax_blood_. However, Held et al. (2024) reported a lower SEM of approximately 0.02 mmol·L^−1^·s^−1^ in trained rowers. Accordingly, an individual change in *v*Lamax_blood_ would need to exceed approximately 0.20 mmol·L^−1^·s^−1^ before it can be distinguished from measurement error. Relative to the observed mean *v*Lamax_blood_ values, this corresponds to a substantial change of approximately 30%–40%, depending on the ergometer system. Smaller changes, even if potentially meaningful from a training perspective, may therefore remain within the expected measurement error.

This contrasts with the mechanical sprint outcomes, which showed substantially lower relative error and are obtained directly from the ergometer without additional blood sampling or calculation steps. Consequently, mechanical power variables may be more suitable for detecting smaller within‐athlete changes with less methodological burden, provided that familiarization has been completed and testing conditions are standardized. By contrast, *v*Lamax_blood_ may be more appropriate for describing broader between‐athlete differences or larger adaptations, whereas small longitudinal changes should be interpreted cautiously. Because high ICC values can coexist with substantial absolute measurement error, absolute indices such as SEM and MDC are particularly important when individual changes are of interest (Atkinson & Nevill, 1998; de Vet et al., 2006; Hopkins, 2000; Warneke et al., 2025). At present, intervention evidence for *v*Lamax_blood_ is scarce, and one of the few available training studies reported no significant between‐group differences in *v*Lamax_blood_ after 6 weeks of sprint‐interval or endurance training despite improvements in calculated power at maximal lactate steady state (Hommel et al., 2019).

The larger error in *v*Lamax_blood_ is methodologically plausible given that the variable is calculated from several components rather than measured directly (Mader, 1984; Wackerhage et al., 2025). It depends on lactate accumulation, sprint duration, and the estimated alactic time component; errors in any of these components can carry over into the final value. The wide limits of agreement for *t*_alac_ point to substantial uncertainty in this component of the *v*Lamax_blood_ calculation. This comparatively low reliability of *t*_alac_ is consistent with previous studies reporting poor to highly variable reliability for the alactic time component, including an ICC of 0.25 in Sablain et al. (2025), while a recent systematic review reported ICC values ranging from −0.12 to 0.88 across different *t*_alac_ estimation approaches and protocols (Röhrs et al., 2026). Since *t*_alac_ is part of the denominator of the *v*Lamax_blood_ equation, small inconsistencies in its estimation may affect the calculated lactate accumulation rate, which is consistent with previous work showing that different *t*_alac_ estimation approaches can influence the reliability of *v*Lamax_blood_ estimates (Meixner et al., 2024; Quittmann et al., 2020).

This also supports recent arguments that alternative lactate‐accumulation metrics without correction for *t*_alac_, such as *v*Lapeak, may provide a more transparent and potentially less error‐prone outcome when the aim is to quantify the observed post‐exercise lactate response rather than to estimate maximal glycolytic rate capacity (Wackerhage et al., 2025). The present findings support this expectation with regard to measurement error. *v*Lapeak showed lower SEM, CV, MAPE, and MDC values than *v*Lamax_blood_ on both ergometer systems. In particular, MDC was approximately 0.11–0.12 mmol·L^−1^·s^−1^ for *v*Lapeak compared with 0.19–0.20 mmol·L^−1^·s^−1^ for *v*Lamax_blood_. However, relative reliability was not consistently superior, as the ICC was higher for *v*Lamax_blood_ on the Lode ergometer but higher for *v*Lapeak on the SRM ergometer. Thus, omission of the *t*_alac_ correction was associated with lower absolute measurement error but did not uniformly improve relative reliability. Nevertheless, MDC remained greater than SWC for *v*Lapeak on both ergometers, indicating that small within‐athlete changes may still be difficult to distinguish from measurement error.

In the present study, post‐exercise lactate was sampled at 1‐min intervals until the highest value was observed. Although this procedure is practical and commonly used, it may miss the exact timing and magnitude of the true lactate peak, particularly when peak lactate occurs between two sampling points. Unlike power output, which is recorded continuously during the sprint, both *v*Lamax_blood_ and *v*Lapeak depend on a delayed physiological response and the correct identification of peak post‐exercise lactate (Goodwin et al., 2007). These factors are consistent with recent review evidence showing substantial heterogeneity in *v*Lamax_blood_ protocols, including sprint duration, ergometer mode, cadence control, warm‐up procedures, lactate analyzers, and calculation approaches (Röhrs et al., 2026).

The use of two ergometer systems increases the practical relevance of the findings and reflects the use of different laboratory‐specific setups in applied performance diagnostics. Reliability indices were therefore reported separately for each system. Relative reliability was not consistently superior for one ergometer across variables. For *v*Lamax_blood_, ICC was higher on the Lode ergometer, whereas *v*Lapeak showed very similar ICC values between systems and *t*_alac_ showed low reliability on both. However, because ergometer allocation was a between‐subject factor, differences between systems should not be interpreted as causal device effects. The separated reporting nevertheless indicated that absolute values and reliability characteristics may differ across ergometers, particularly for variables involved in the *v*Lamax_blood_ calculation. The comparatively long *t*_alac_ values observed on the Lode ergometer should also be interpreted cautiously. Previous studies using the Lode Excalibur Sport in isokinetic mode at 130 rpm reported a mean time to peak power of 4.31 ± 0.70 s (Haase et al., 2024). However, these comparisons are based on different participant samples and therefore do not allow device‐specific effects to be isolated. Differences in *t*_alac_ may reflect ergometer characteristics, between‐sample differences, or an interaction between both. Future studies should directly compare ergometer systems within the same participants to determine whether systematic device‐related differences in time to peak power and derived *v*Lamax_blood_ values exist. Therefore, results obtained from different ergometer systems should not be used interchangeably. Longitudinal monitoring should be performed on the same ergometer using identical cadence control, device settings, sampling frequency, lactate sampling procedures, and data processing.

### 4.1 Practical applications

The present findings have direct implications for sprint‐based diagnostics. Mechanical sprint outcomes appear suitable for longitudinal monitoring after familiarization, provided that testing conditions are standardized. In contrast, *v*Lamax_blood_ should be interpreted more cautiously at the individual level, and changes should clearly exceed approximately 0.20 mmol·L^−1^·s^−1^ before being considered likely true physiological adaptations. *v*Lapeak showed lower measurement error than *v*Lamax_blood_ and may therefore provide a more precise measure of lactate accumulation when correction for *t*_alac_ is not required. This gain in precision should, however, be interpreted in light of the simpler calculation underlying *v*Lapeak, which does not incorporate the additional information represented by the *t*_alac_ correction. Accordingly, the two metrics should be considered related but not necessarily interchangeable. Future studies should determine to what extent *v*Lapeak and *v*Lamax_blood_ reflect the same underlying physiological characteristics across different populations and sprint protocols. Because MDC remained greater than SWC, small individual changes in *v*Lapeak should also be interpreted cautiously.

### 4.2 Limitations

Some limitations should be considered. The sample was recruited on an ad hoc basis and consisted of active athletes without specific cycling experience. This was appropriate for the study aim, but the results may not transfer directly to trained cyclists, sprint specialists, or athletes already familiar with cycle‐ergometer sprint testing. Although the total sample size met the a priori target, the device‐specific reliability analyses were based on smaller subgroups (Lode: *n* = 17; SRM: *n* = 33) and therefore provide less precise estimates, particularly for the Lode ergometer. Furthermore, the stationary start and potential delay in force application on the Lode ergometer may have prolonged the acceleration phase required to reach the target cadence of 130 rpm, resulting in higher *t*_alac_ values and consequently higher calculated *v*Lamax_blood_ values. Variability in this effect may have introduced additional measurement error and reduced the reliability of *v*Lamax_blood_. The study included three test sessions, allowing short‐term reliability and systematic changes to be examined, but it remains unclear whether further familiarization would have led to additional stabilization. Moreover, only one 15‐s sprint protocol was examined; other sprint durations, ergometer settings, sampling intervals, or methods for estimating the alactic contribution may produce different reliability characteristics. Hydration status was not controlled across sessions, which may have contributed to variability in blood lactate concentrations and affected their test–retest reliability. Because sprint duration was fixed at 15 s, *v*Lapeak represents Δ lactate scaled to the complete sprint duration; therefore, its reliability characteristics are inherently linked to those of Δ lactate. Its lower measurement error relative to *v*Lamax_blood_ should consequently be interpreted primarily as an effect of omitting the variable *t*_alac_ correction. Future studies should examine whether additional familiarization sessions, repeated baseline trials, stricter pre‐test control, or modified *t*_alac_ estimation improve the reproducibility and individual‐level interpretation of *v*Lamax_blood_.

## 5 CONCLUSION

In conclusion, mechanical sprint variables showed high test–retest reliability and comparatively low absolute error, with ICC values ranging from 0.926 to 0.995 and CV values from 1.75% to 4.39%. However, peak and mean power increased systematically across repeated sessions, with significant pairwise effects ranging from *d*z = 0.475 to 0.611, indicating that familiarization should be considered before baseline testing in athletes without specific cycling experience. Accordingly, the first sprint test should not be used as the sole baseline assessment, and at least one familiarization session appears advisable. In contrast, *v*Lamax_blood_ showed lower individual‐level precision, with MAPE values of 14.78% to 15.35% and MDC values of approximately 0.19–0.20 mmol·L^−1^·s^−1^. Accordingly, small within‐athlete changes in *v*Lamax_blood_ should be interpreted cautiously unless they clearly exceed the expected measurement error. *v*Lapeak showed good relative reliability and lower measurement error than *v*Lamax_blood_, with MDC values of approximately 0.11–0.12 mmol·L^−1^·s^−1^. However, relative reliability was not uniformly higher, and MDC remained greater than SWC, indicating that small individual changes in *v*Lapeak should also be interpreted cautiously. The comparatively low reliability of *t*_alac_ further indicates that the estimation of the alactic time component is a relevant source of uncertainty in calculated *v*Lamax_blood_. Because absolute values and reliability characteristics differed descriptively between ergometer groups, longitudinal assessments should be performed on the same ergometer with identical cadence control, device settings, data processing, and lactate sampling procedures. Overall, these findings highlight the need to distinguish between directly measured mechanical outcomes and calculated lactate‐accumulation metrics when using sprint‐based diagnostics for longitudinal performance monitoring.

## AUTHOR CONTRIBUTIONS

**Daniel Röhrs:** Conceptualization; data curation; formal analysis; investigation; methodology; project administration; visualization. **Carl‐Maximilian Wagner:** Conceptualization; methodology. **Nicolas Neumann:** Investigation. **Rüdiger Reer:** Supervision. **Tobias Schmidt:** Conceptualization; methodology; supervision. **Michael Keiner:** Conceptualization; methodology; supervision.

## FUNDING INFORMATION

We acknowledge financial support from the Open Access Publication Fund of Universität Hamburg.

## CONFLICT OF INTEREST STATEMENT

The authors report there are no competing interests to declare.

## ETHICS STATEMENT

The study was conducted in accordance with the Declaration of Helsinki and was approved by the local university ethics committee (MSH‐2023/287, 18 September 2023).

## CONSENT

All participants provided written informed consent prior to participation.

## Data Availability

The data that support the findings of this study are available from the corresponding author upon reasonable request. *Code Availability Statement*: The analysis code used to support the findings of this study is available from the corresponding author upon reasonable request.
